# Maternal and neonatal vitamin D metabolite profiling and its long-term impact on childhood growth: findings from the KLOTHO birth cohort

**DOI:** 10.3389/fendo.2026.1772187

**Published:** 2026-03-25

**Authors:** Spyridon N. Karras, Maria Kypraiou, Vikentia Harizopoulou, Antonios Vlastos, Marios Anemoulis, Maria Dalamaga, Neoklis Georgopoulos, Evanthia Kassi, Georgios Mastorakos, Kali Makedou, Dimitrios Skoutas, Melpomeni Peppa, Dimitrios G. Goulis

**Affiliations:** 1 Laboratory of Biological Chemistry, Medical School, Aristotle University, Thessaloniki, Greece; 2 Assisting Nature Centre of Reproduction and Genetics, Thessaloniki, Greece; 3 Department of Midwifery, Faculty of Health and Caring Sciences, University of West Attica, Athens, Greece; 4 Department of Biological Chemistry, National and Kapodistrian University of Athens, Athens, Greece; 5 Division of Endocrinology, Department of Internal Medicine, School of Health Sciences, University of Patras, Patras, Greece; 6 Second Department of Surgery, Medical School, Aretaieio Athens Hospital, National and Kapodistrian University of Athens, Athens, Greece; 7 Laboratory of Biochemistry, American Hellenic Educational Progressive Association Hospital (AHEPA) University Hospital, School of Medicine, Aristotle University of Thessaloniki, Thessaloniki, Greece; 8 Thermi Clinic, Thessaloniki, Greece; 9 Endocrine Unit, 2ndPropaedeutic Department of Internal Medicine, Attikon University Hospital, Medical School, National and Kapodistrian University of Athens, Athens, Greece; 10 Unit of Reproductive Endocrinology, 1stDepartment of Obstetrics and Gynecology, Medical School, Faculty of Health Sciences, Aristotle University of Thessaloniki, Thessaloniki, Greece

**Keywords:** children growth, KLOTHO birth cohort, metabolites, vitamin (25[OH]D), vitamin D

## Abstract

**Background:**

Vitamin D is increasingly recognized as a key modulator of growth, metabolism, and body composition in early life. However, the long-term impact of maternal vitamin D status and its multiple circulating forms on childhood anthropometry remains poorly understood. The KLOTHO cohort provides a unique opportunity to investigate these associations using detailed multi-form vitamin D profiles.

**Methods:**

Within the prospective KLOTHO cohort, serum concentrations of eight vitamin D metabolites [25(OH)D_2_, 25(OH)D_3_, 1α,25(OH)_2_D_2_, 1α,25(OH)_2_D_3_, 3-epi-25(OH)D_2_, 3-epi-25(OH)D_3_, D_2_, D_3_] were quantified at birth by liquid chromatography–tandem mass spectrometry (LC–MS/MS). Anthropometric measurements were assessed at 10–11 years of age (height, weight, BMI, waist circumference, and skinfold thickness). Associations between log_10_-transformed metabolite levels and anthropometric outcomes were evaluated using Spearman’s correlation and multivariable linear regression adjusted for available covariates (sex, birth weight, maternal BMI, and season). False discovery rate (FDR) correction was applied (q <0.10).

**Results:**

Among 98 children with available follow-up data, cord-blood vitamin D metabolite profiles showed several exploratory trends of association with anthropometric measures assessed at 10–11 years of age. Directionally consistent associations were observed primarily for D_3_-related metabolites and linear growth indices, as well as for selected adiposity-related measures. However, none of the observed associations demonstrated robust statistical significance after correction for multiple testing. All findings should therefore be interpreted as hypothesis-generating signals rather than confirmed long-term associations.

**Conclusions:**

In this exploratory analysis, multidimensional profiling of vitamin D metabolites at birth identified preliminary trends linking D_3_-related metabolites with later childhood anthropometric measures. These findings are hypothesis-generating and underscore the need for larger, adequately powered longitudinal studies to clarify the role of early-life vitamin D metabolism in childhood growth.

## Introduction

Vitamin D is an essential secosteroid hormone with pleiotropic functions extending beyond bone metabolism. It plays a crucial role in immune regulation, muscle development, and energy homeostasis, influencing multiple pathways that contribute to long-term metabolic health (1, 2). Evidence from experimental and clinical studies indicates that vitamin D status during pregnancy has been associated with fetal growth and adiposity, with implications that extend into childhood (3). Maternal vitamin D deficiency has been associated with altered birth weight, reduced bone mineral accrual, and an increased risk of obesity and insulin resistance in later life (4–6). Despite the growing recognition of these associations, most studies in humans have relied on quantification of a single vitamin D metabolite, 25-hydroxyvitamin D [25(OH)D]. However, vitamin D metabolism is complex and involves hydroxylation, epimerization, and conjugation steps that generate multiple biologically active and inactive compounds (7). These include the parent forms D_2_ and D_3_, their hydroxylated metabolites and their epimers [3-epi-25(OH)D_2_, 3-epi-25(OH)D_3_], which may possess distinct receptor affinities and biological effects (8). Analytical advances, particularly liquid chromatography–tandem mass spectrometry (LC–MS/MS), now allow the simultaneous measurement of these metabolites, providing a complete view of vitamin D metabolism and its physiological implications (9–14).

The KLOTHO cohort is a prospective mother–child study conducted in Northern Greece (2013–2015), designed to investigate endocrine and metabolic determinants of early-life health, with cord blood sampling at birth and longitudinal anthropometric follow-up in childhood.

In this analysis, we examined whether vitamin D metabolite profiles at birth were associated with anthropometric outcomes assessed in late childhood. Given the limited existing data on multi-metabolite vitamin D profiling in developmental cohorts, the present analysis was exploratory and hypothesis-generating.

## Methods

### Study design and participants

The KLOTHO study is a longitudinal, observational cohort study that investigates the endocrine, metabolic, and nutritional determinants of health across the maternal–child axis. Pregnant women were recruited during the third trimester from outpatient obstetric clinics in Northern Greece between 2013 and 2015. The inclusion criteria were singleton pregnancies, absence of chronic diseases, and availability of cord serum samples at delivery. The exclusion criteria were preterm birth (<37 weeks), gestational diabetes, and chronic corticosteroid use. For the present analysis, data from 98 mother–child pairs with complete anthropometric evaluations at 10–11 years of age were included. Ethical approval was granted by the Bioethics Committee of the Medical School, Aristotle University of Thessaloniki, Greece and written informed consent was obtained from all participants or their legal guardians, in accordance with the Declaration of Helsinki.

### Biochemical measurements

Umbilical cord serum samples collected at birth were stored at –80 °C until analysis. Eight vitamin D metabolites were quantified using LC–MS/MS: 25(OH)D_2_, 25(OH)D_3_, 1α,25(OH)_2_D_2_, 1α,25(OH)_2_D_3_, 3-epi-25(OH)D_2_, 3-epi-25(OH)D_3_, D_2_, and D_3_. The method demonstrated inter-assay coefficients of variation below 8% and lower limits of detection <1 nmol/L for all the analytes. Calibration was performed using certified reference materials (NIST SRM 972a). For analytical stability, all samples were processed in duplicate, and batch effects were minimized by the inclusion of internal quality controls.

### Anthropometric assessment at 10–11 years of age

Anthropometric measurements were performed by trained personnel following standardized pediatric anthropometric protocols, with duplicate measurements averaged when available. Measurements included body weight (kg), height (cm), waist circumference (cm), mid-upper arm circumference (cm), and skinfold thickness (mm) (triceps and subscapular) and lower waist circumference refers to the minimal abdominal circumference measured. Knee–heel length was measured using a standardized anthropometric method as an additional proxy of lower limb growth. Measurements were performed according to standardized pediatric anthropometric protocols based on established WHO guidelines, thereby improving methodological transparency and reproducibility. The body mass index (BMI, kg/m²) was calculated and converted to age- and sex-specific percentiles using WHO reference data. Height and weight were analyzed as continuous variables, whereas BMI percentiles were used to account for age- and sex-related growth variability. Body fat percentage was estimated using bioelectrical impedance (Tanita BC-420, Japan).

### Statistical analysis

All analyses were conducted in Python (v3.11) using the pandas, statsmodels, and scipy libraries. Vitamin D metabolite concentrations were log_10_-transformed, and non-positive values were imputed as half the smallest positive value per analyte. Pairwise associations between metabolites and anthropometric indices were assessed using Spearman’s ρ with Benjamini–Hochberg false discovery rate (FDR) adjustment (q <0.10 was considered significant). Multivariable linear regressions were performed for each anthropometric outcome, including log10-transformed metabolites as independent variables, and adjusted for sex, birth weight, maternal BMI, and season of birth (used as a surrogate marker of UVB exposure). Outcomes were standardized to z-scores to yield comparable β coefficients. Sensitivity analyses excluded extreme outliers [>3 standard deviations (SD)]. Analyses were two-tailed with α = 0.05 level of significance. Given the exploratory nature of the study and the limited sample size, an FDR threshold of q < 0.10 was used to reduce the risk of overlooking potentially relevant signals, while acknowledging that no associations survived this correction. Imputed values represented less than 5% of measurements per metabolite. Given the sample size and number of comparisons, all analyses were considered exploratory and hypothesis-generating.

## Results

The original KLOTHO cohort included 144 mother–child pairs at baseline. Of these, 46 were excluded or lost to follow-up according to predefined criteria, resulting in 98 mother–child pairs available for the present analysis.

At the 10–11-year follow-up, detailed anthropometric data were available for the cohort (N = 98; 51 girls, 47 boys). Outcome-specific sample sizes are indicated in **Tables 1**–**3**. Analyses based on smaller subsamples (N < 25) are reported for completeness but should be interpreted cautiously due to limited statistical power.

**Table 1:** Maternal, paternal, and child anthropometric characteristics.

Variable	N	Mean ± SD	Median [IQR]
BMI pre-pregnancy (kg/m^2^)	71	22.8 ± 3.7	22.2 [20.1–24.6]
BMI term (kg/m^2^)	71	27.6 ± 3.9	27.00 [24.9–29.9]
Birth weight (g)	66	3267 ± 364	3220 [3010–3515]
Educational level	68	2.04 ± 0.76	2.00 [1.00–3.00]
Maternal Height (cm)	71	166.3 ± 6.4	166.0 [162.0–171.5]
Paternal Height (cm)	12	175.1 ± 5.7	175.0 [170.8–179.0]
Knee-heel length (cm)	25	8.70 ± 0.80	8.90 [8.20–9.30]
Season	71	1.32 ± 0.47	1.00 [1.00–2.00]
Sex	70	1.63 ± 0.49	2.00 [1.00–2.00]
UVB (Wh/m²)-45 d	59	0.20 ± 0.11	0.22 [0.10–0.31]
Weight pre-pregnancy (kg)	71	63.1 ± 10.8	62.0 [55.0–68.0]
Weight term (kg)	71	76.4 ± 12.0	74.0 [68.5–83.0]
Child’s height	21	134.1 ± 10.8	136.0 [125.0–142.0]
Lower waist circumference (cm)	20	70.8 ± 18.2	76.0 [63.8–81.3]
Sun exposure summer daily	8	5.00 ± 1.60	4.50 [4.00–6.00]
Weight (kg)	21	32.6 ± 9.5	32.0 [27.0–40.0]

BMI, body mass index; IQR, interquartile range; SD, standard deviation; UVB, ultraviolet B. Anthropometric outcomes with N < 25 were retained for exploratory purposes only and should be interpreted cautiously. Waist circumference and lower waist circumference represent different standardized abdominal measurement sites. Denotes technical replicate measurement from LC–MS/MS analysis.

**Table 2:** Selected correlations between vitamin D metabolites and anthropometric or maternal variables.

Outcome	Metabolite (nmol/L)	rho	p	N	q_FDR
Knee-heel length (cm)	25(OH)D_2_	-0.635	0.001	21	0.1124
Educational level	3-epi-25(OH)D_2_.1	0.423	0.001	47	0.1124
Knee-heel length (cm)	25(OH)D_2_	-0.594	0.002	35	0.1133
Educational level	Total 3-epi-25(OH)D	0.397	0.003	47	0.1200
Educational level	3-epi-25(OH)D_2_	0.388	0.003	48	0.1226
Sex	25(OH)D_2_	0.325	0.006	56	0.1992
Season	1α,25(OH)_2_D_3_	0.344	0.008	56	0.2132
UVB (Wh/m²)-45 d	Total 3-epi-25(OH)D.1	-0.329	0.011	48	0.2678
UVB (Wh/m²)-45 d	Total 3-epi-25(OH)D	-0.323	0.013	47	0.2713
Season	epi-25(OH)D_3_	0.279	0.019	45	0.3558
Educational level	25(OH)D_2_	0.281	0.020	67	0.3558
Birth weight (g)	25(OH)D_3_.	-0.266	0.031	78	0.4586
Height (cm)	3-epi-25(OH)D_2_.	0.280	0.032	79	0.4586
Season	D_3_	-0.275	0.035	68	0.4586
Knee-heel length (cm)	epi-25(OH)D_3_	-0.423	0.035	24	0.4586
UVB (Wh/m²)-45 d	3-epi-25(OH)D_2_	-0.267	0.041	38	0.4606
BMI pre-pregnancy (kg/m2)	25(OH)D_2_	-0.243	0.041	79	0.4606
Educational level	D_3_	-0.271	0.043	57	0.4606
Height (cm)	D_3_	-0.279	0.045	89	0.4606
UVB (Wh/m²)-45 d	3-epi-25(OH)D_2_	-0.246	0.060	68	0.5608
UVB (Wh/m²)-45 d	D_2_	-0.23	0.079	57	0.5862
Educational level	epi-25(OH)D_3_	0.211	0.085	68	0.5862
Educational level	1α,25(OH)_2_D_3_	0.232	0.085	35	0.5862
UVB (Wh/m²)-45 d	epi-25(OH)D_3_	-0.222	0.091	49	0.5862
Birth weight (g)	Total 3-epi-25(OH)D	0.232	0.091	67	0.5862
Educational level	epi-25(OH)D_3_	0.207	0.091	46	0.5862
UVB (Wh/m²)-45 d	1α,25(OH)_2_D_3_	-0.220	0.094	47	0.5862
Educational level	Total 3-epi-25(OH)D	0.220	0.104	57	0.5862
Weight pre-pregnancy (kg)	D_2_	-0.213	0.106	57	0.5862
Sex	D_2_	-0.211	0.109	56	0.5862
Season	Total 3-epi-25(OH)D	0.210	0.110	58	0.5862
Sex	D_3_	-0.210	0.111	49	0.5862
UVB (Wh/m²)-45 d	epi-25(OH)D_3_	-0.204	0.121	45	0.6199
Child’s height	epi-25(OH)D_3_	0.342	0.130	48	0.6484
lower waist circumference (cm)	25(OH)D_2_	0.318	0.172	56	0.7036
child’s height	25(OH)D_2_	0.229	0.318	46	0.7878
weight(kg)	25(OH)D_2_	0.216	0.346	67	0.7878
lower waist circumference (cm)	epi-25(OH)D_3_	0.222	0.347	56	0.7878
weight(kg)	epi-25(OH)D_3_	0.208	0.365	56	0.7915

BMI, body mass index; FDR, false discovery rate; IQR, interquartile range; SD, standard deviation; UVB, ultraviolet B. No associations remained statistically significant after false discovery rate (FDR) correction. ”.1” denotes technical replicate measurement from LC–MS/MS analysis.

**Table 3:** Adjusted regression models (β, SE, p, q_FDR).

Outcome	Metabolites (nmol/L)	Beta_std	SE	p	N	q_FDR
Sex	25(OH)D_2_	0.656	0.206	0.002	70	0.165
Educational level	3-epi-25(OH)D_2_	0.565	0.177	0.002	56	0.165
Educational level	25(OH)D_2_	0.645	0.209	0.003	68	0.165
Educational level	Total 3-epi-25(OH)D	0.493	0.167	0.005	56	0.190
Educational level	3-epi-25(OH)D_2_	0.421	0.15	0.007	56	0.227
Height (cm)	3-epi-25(OH)D_2_	0.468	0.177	0.011	59	0.295
Birth weight (g)	Total 3-epi-25(OH)D	0.564	0.232	0.019	54	0.442
Child Height (cm)	D_2_	0.648	0.276	0.023	52	0.458
Weight term (kg)	25(OH)D_2_	-0.483	0.211	0.025	71	0.458
UVB (Wh/m²)-45 d	D_2_	-0.521	0.242	0.035	59	0.527
Season	1α,25(OH)_2_D_3_	1.998	0.928	0.036	59	0.527
Child Height (cm)	D_3_	0.557	0.264	0.039	59	0.527
BMI term (kg/m^2^)	25(OH)D_2_	-0.435	0.212	0.044	71	0.527
BMI pre-pregnancy (kg/m^2^)	25(OH)D_2_	-0.403	0.203	0.050	71	0.527
Sex	D_3_	-0.529	0.265	0.051	59	0.527
Season	D_3_	-0.523	0.265	0.054	59	0.527
Birth weight (g)	D_2_	0.506	0.267	0.063	54	0.575
Educational level	D_3_	-0.508	0.271	0.066	56	0.575
UVB (Wh/m²)-45 d	Total 3-epi-25(OH)D	-0.303	0.171	0.082	59	0.587

BMI, body mass index; FDR, false discovery rate; SE, standard error; UVB, ultraviolet B. No associations remained statistically significant after false discovery rate (FDR) correction. ”.1” denotes technical replicate measurement from LC–MS/MS analysis.

The availability of anthropometric variables varied across outcomes; height and BMI were available for most participants, whereas waist circumference, weight, and knee–heel length were available in smaller subsamples (N = 20–25).The mean age at the time of examination was 10.6 ± 0.3 years. Standing height averaged 142.8 ± 7.6 cm, while body weight was 32.6 ± 9.5 kg based on the subset with available data (N = 21). The mean BMI was 18.9 ± 2.8 kg/m², corresponding to a BMI percentile of 53 ± 18 according to the WHO Health Organization growth charts, indicating a population comparable to population-based estimates for children of similar age. Measures of body composition showed a mean triceps skinfold thickness of 13.4 ± 3.2 mm and a mean body fat percentage of 23.5 ± 6.9%. Knee–heel length (N = 25), an additional proxy for linear growth, averaged 8.70 ± 0.80 cm. No statistically significant sex differences were detected for any of the anthropometric parameters after adjusting for age (p > 0.10). These values are presented descriptively in (**Table 1**). Spearman correlation analyses (**Table 2**) revealed several unadjusted associations between vitamin D metabolites and maternal or child characteristics, although most were not significant after false discovery rate (FDR) correction. The strongest associations were observed between 25(OH)D_2_ and knee–heel length, showing a moderate negative correlation (ρ = –0.635, p = 0.0006, q = 0.112), and similarly for the duplicate measurement (ρ = –0.594, p = 0.0017, q = 0.113). Epimeric metabolites demonstrated positive correlations with maternal educational level, including 3-epi-25(OH)D_2_ (ρ = 0.423, p = 0.0012) and total epimers (ρ = 0.397, p = 0.0025), although they failed to meet the FDR-adjusted significance thresholds. Associations with season and UVB exposure were also noted; for example, 1α,25(OH)_2_D_3_ correlated with season (ρ = 0.344, p = 0.0077), and total epimers correlated inversely with ultraviolet B (UVB) (ρ = –0.329, p = 0.011). These correlations describe associations between metabolites and environmental or maternal variables; causal interpretation is not possible within this design.

**Figure 1:**
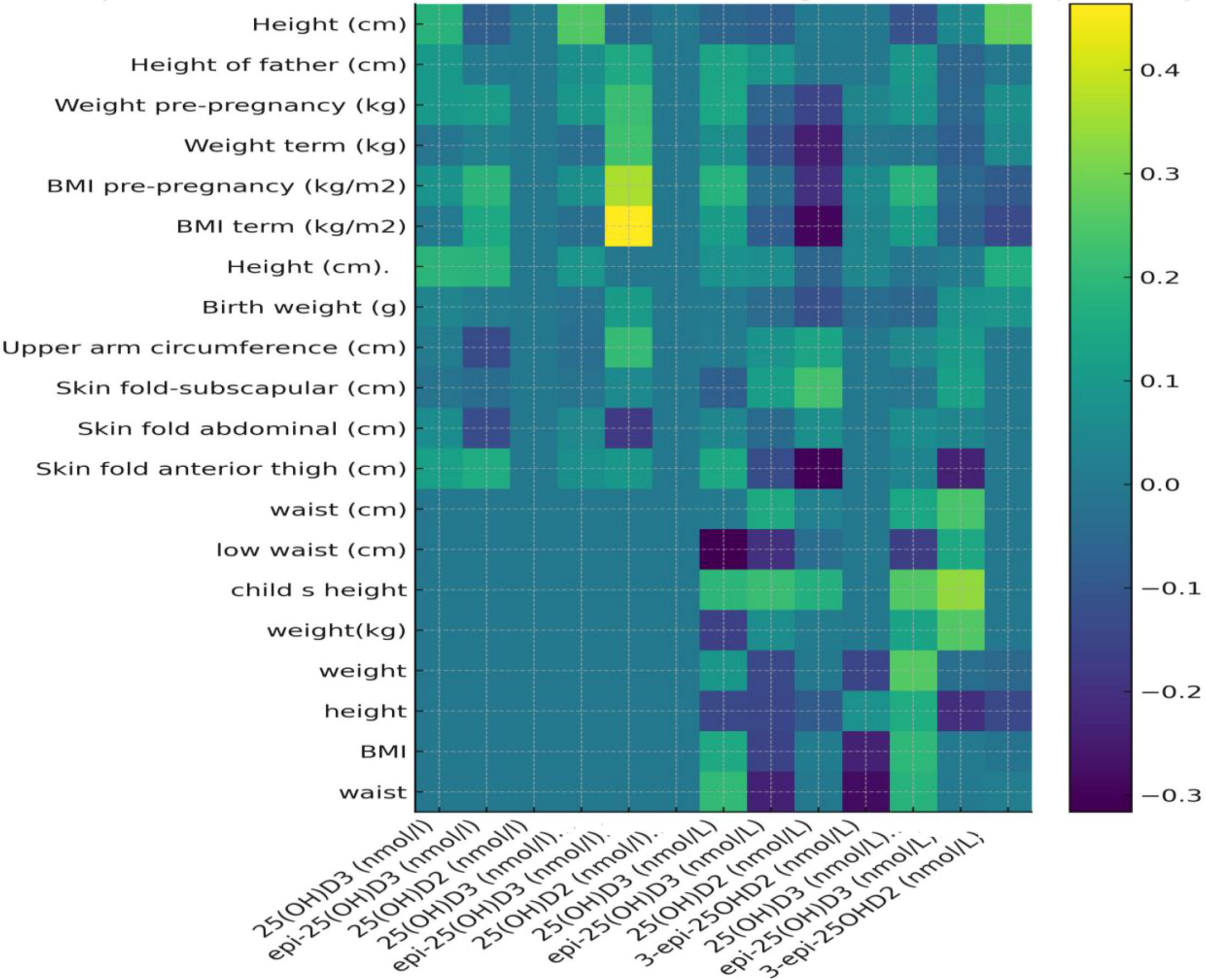
Heatmap of Spearman’s ρ coefficients between vitamin D metabolites quantified at birth (log_10_) and anthropometric indices at 10–11 years of age. Positive Spearman’s ρ coefficients are shown in yellow, whereas negative associations are shown in purple. Color intensity reflects the magnitude of the correlation. Sample size varies across outcomes and is reported in the corresponding tables.

**Figure 2:**
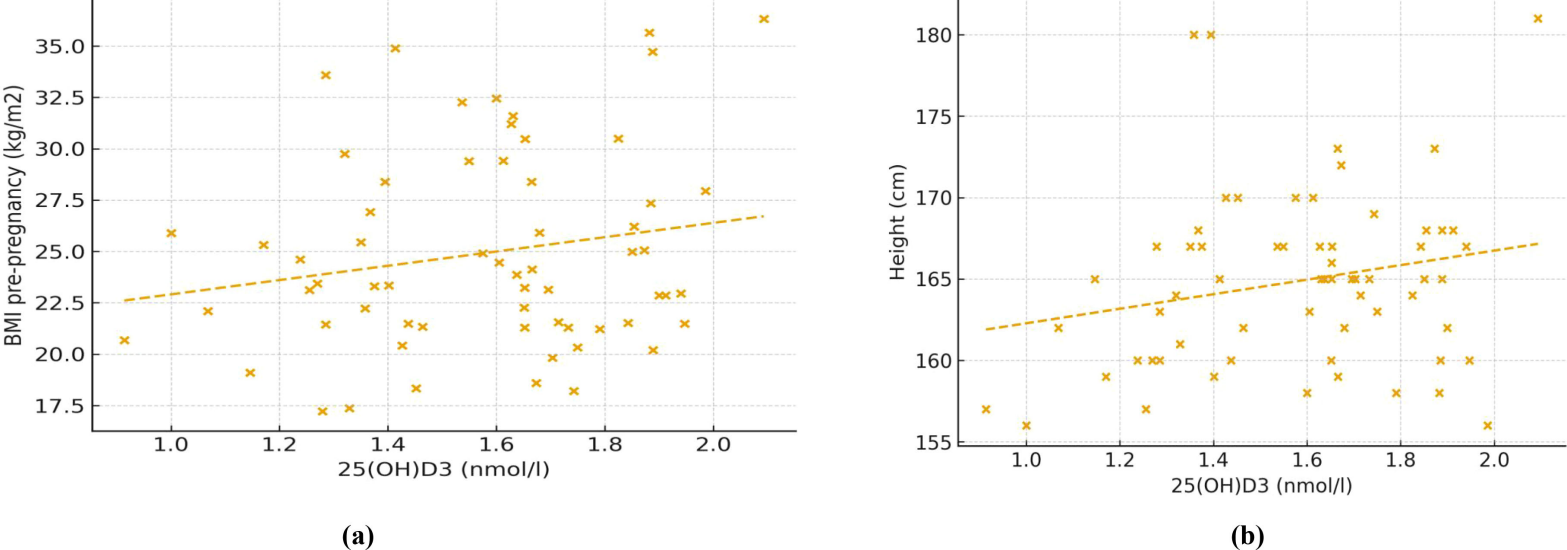
Illustration of the direction and magnitude of standardized regression coefficients for exploratory associations between 25(OH)D_3_ and **(A)** BMI and **(B)** height. Standardized β coefficients and 95% confidence intervals from multivariable models are shown. Associations did not survive correction for multiple testing and are presented for visualization purposes only. Regression models are based on complete-case analyses; sample size for each model is indicated in **Table 3**.

Regarding child anthropometry, several vitamin D metabolites were associated with linear growth. Although none of these associations remained significant after FDR correction, their directionality is reported here for completeness as exploratory findings. For instance, 3-epi-25(OH)D_2_ correlated with height (ρ = 0.28, p = 0.032), whereas Vitamin D_3_ (nmol/L) showed a positive trend (ρ = 0.252, p = 0.054). Scatterplots (**Figures 1**, **2**) illustrate the distribution and direction of regression coefficients derived from exploratory analyses. No significant correlations were observed between vitamin D metabolite levels and weight, BMI, or waist circumference, although some weak inverse associations appeared for adiposity parameters [e.g., 25(OH)D_3_ with low waist: ρ = –0.275, p = 0.240]. Overall, correlation analyses indicated that D_3_-related metabolites were more frequently associated with linear growth indices than D_2_-derived metabolites.

In multivariable linear regression models adjusted for sex, birth weight, maternal BMI, and season (**Table 3**), several metabolites remained associated with child outcomes. 3-epi-25(OH)D_2_ showed a positive standardized effect on standing height (β = 0.468, SE = 0.177, p = 0.0107, q = 0.295), consistent with the correlation findings. Vitamin D_3_ also demonstrated a positive adjusted association with height (β = 0.557, SE = 0.264, p = 0.0393). Additionally, the total epimers predicted a higher birth weight (β = 0.564, SE = 0.232, p = 0.0187). D_2_ metabolites displayed inconsistent patterns, including negative associations with maternal weight at term (β = –0.483, p = 0.025) and BMI (β = –0.435, p = 0.044). Notably, none of the regression associations survived the FDR correction, reflecting the modest sample sizes available for each model (N = 52–71).Sample size varied across regression models due to complete-case analysis for each outcome. Despite these observations, directional consistency alone does not constitute evidence of a biological effect in the absence of statistically robust findings.

## Discussion

The present study examined the long-term associations between a comprehensive panel of vitamin D metabolites measured in cord blood and anthropometric outcomes at 10–11 years of age in the KLOTHO cohort. Using LC–MS/MS quantification of eight metabolites, including parent forms, hydroxylated derivatives, and epimers, we evaluated both unadjusted correlations and adjusted regression models to identify metabolic patterns that may contribute to childhood growth and adiposity.

In this exploratory analysis, several vitamin D metabolites showed directionally similar associations with anthropometric measures in late childhood; however, none of these associations remained statistically significant after correction for multiple testing. The findings should therefore be interpreted as preliminary signals rather than evidence of robust or consistent effects. Accordingly, the present results are hypothesis-generating and require confirmation in larger, adequately powered cohorts. These findings are biologically plausible in light of existing literature on early-life vitamin D exposure; however, they do not provide evidence for fetal metabolic programming effects.

Children in this cohort demonstrated anthropometric values largely representative of the general population, with a mean BMI of 18.9 kg/m² and body fat percentage of 23.5%. Against this relatively narrow distribution of growth parameters, we observed modest yet directionally coherent associations linking higher D_3_-derived metabolite levels at birth with taller stature at follow-up. Specifically, 25(OH)D_3_ and its epimer displayed positive coefficients for height in the multivariable models, while the raw correlations showed similar trends. Although several associations showed similar directions across unadjusted and adjusted analyses, directional consistency alone does not provide statistical evidence in the absence of associations surviving correction for multiple testing. Accordingly, these observations should be interpreted strictly as exploratory trends rather than as biologically confirmed effects.

Inverse associations between D_3_ metabolites and adiposity markers were also evident in the unadjusted analyses, particularly for BMI and skinfold measurements. Although these did not reach adjusted significance, the directionality is noteworthy, given the experimental evidence that vitamin D modulates adipocyte differentiation, inflammatory tone, and energy metabolism. The pattern observed here (higher D_3_ exposure associated with leaner body composition) mirrors the findings from both neonatal and childhood cohorts (15–18), reporting that prenatal vitamin D insufficiency predicts increased adiposity and altered metabolic risk in offspring (19–21).

In contrast, D_2_-derived metabolites did not show meaningful or biologically coherent associations with childhood anthropometry. Most observed correlations with D_2_ metabolites involved maternal characteristics (e.g., maternal BMI and educational level) rather than child outcomes (**Tables 2**, **3**). This likely reflects the external, dietary-dominant nature of vitamin D_2_ exposure in this population, as well as its lower affinity for vitamin D-binding protein and the vitamin D receptor (VDR), resulting in weaker physiological relevance compared with D_3_ (22). The absence of clear D_2_–anthropometry associations reinforces the differential biological impact of the two vitamin D pathways and suggests that total 25(OH)D measurements may mask metabolite-specific relationships.

Several exploratory associations were observed between epimeric vitamin D metabolites and selected anthropometric or maternal variables. However, these associations did not survive correction for multiple testing and were more frequently observed with maternal or environmental characteristics than with child anthropometric outcomes (**Tables 2**, **3**).

Epimers remain an underexplored area in human developmental studies, despite evidence that they may exhibit distinct receptor affinities and biological activities (23).

Recent experimental studies suggest that epimeric vitamin D metabolites may arise from placental and fetal enzymatic activity and could reflect differential handling of vitamin D during late gestation (24, 25). While their biological activity remains incompletely understood, emerging evidence supports their potential role as indicators of maternal–fetal vitamin D exposure rather than direct mediators of growth-related programming (26).

Epimers may primarily reflect maternal–fetal vitamin D exposure patterns rather than causal mediators of childhood growth and should be interpreted as exposure biomarkers in observational settings (27).

Associations involving epimeric vitamin D metabolites were more frequently observed with maternal or environmental variables, such as education level, season, and UVB exposure. These findings suggest that epimers may primarily reflect exposure-related patterns rather than direct biological effects on childhood anthropometry. Accordingly, epimer-related results should be interpreted as exploratory and hypothesis-generating. Future studies employing mediation models or isotopic tracing could help clarify the contribution of epimers to fetal endocrine development.

A central limitation of this analysis arises from the substantial variation in the sample size across anthropometric measures. While height and BMI were available for approximately 70 children, measures such as waist circumference, mid-upper arm circumference, and knee–heel length were available for only 20–25 participants (**Table 1**). This heavily reduced the power to detect meaningful correlations and led to inflated correlation coefficients in some cases (for example, ρ values exceeding |0.60| for variables with a very small N in **Table 2**). These values should not be interpreted as strong biological effects; rather, they reflect statistical instability due to the limited sample size. The adjusted models partially mitigated this issue but similarly suffered from reduced power when the number of complete cases per model was small. The substantial variability in sample size across anthropometric outcomes represents an important limitation of this study. Outcomes with limited data availability are particularly prone to unstable correlation and regression estimates, and the corresponding results should be viewed as preliminary. Several correlations and regression coefficients were derived from small subsamples. These estimates should be interpreted cautiously, as small sample sizes increase the risk of statistical instability and imprecise effect size estimation. Maternal vitamin D concentrations were not included as covariates, as measurements were not available at comparable time points with harmonized methodology for all participants; inclusion would have markedly reduced the analyzable sample size.

Measurement heterogeneity may also contribute to the absence of robust associations between adiposity indices. Skinfold measurements, although valuable, are operator-dependent and may introduce non-differential measurement errors that bias associations toward the null. Bioimpedance-based body fat estimation is similarly influenced by hydration status and may lack sensitivity in detecting subtle differences among children with predominantly normal BMI.

Despite these challenges, this study makes several important contributions. First, it is among the few longitudinal cohorts to characterize a complete panel of vitamin D metabolites, including epimers, at birth and link them with anthropometry a decade later. This comprehensive metabolic profile enables a nuanced interpretation of the vitamin D system that moves beyond the traditional reliance on total 25(OH)D. Second, the use of LC–MS/MS ensures high analytical precision and accurate differentiation of overlapping metabolites, which is a key requirement in epimer research. Third, by integrating maternal, environmental, and seasonal variables, this study situates vitamin D metabolism within its broader ecological context, highlighting the relevance of endogenous D_3_ pathways over exogenous D_2_ inputs.

Although D_3_-related metabolites showed directionally similar associations across several analyses, directional consistency alone does not constitute evidence of a biological effect in the absence of statistically robust findings. Accordingly, the present results do not support conclusions regarding fetal metabolic programming but rather highlight areas for future hypothesis-driven investigation.

This study has several limitations that must be acknowledged. The modest sample size, particularly for certain anthropometric variables, limits the statistical power and complicates the interpretation of the correlation coefficients. Residual confounding by unmeasured factors such as maternal diet, placental function, or genetic variants in vitamin D metabolism cannot be excluded. The observational design precludes any causal inferences. Finally, the absence of FDR-significant associations requires the discussion to frame all findings as exploratory. Information on pubertal stage was not available at follow-up; therefore, residual confounding related to pubertal timing cannot be excluded. In this exploratory analysis, multidimensional profiling of vitamin D metabolites at birth identified preliminary trends linking D_3_-related metabolites with later childhood anthropometry. These findings are hypothesis-generating and require confirmation in larger cohorts with harmonized anthropometric assessments and adequate statistical power.

In addition, substantial inter-individual variability in both vitamin D metabolite concentrations and anthropometric measures may have attenuated detectable associations. Given the modest subgroup sizes for several outcomes, some analyses were likely underpowered, and effect estimates should be interpreted within the context of exploratory, hypothesis-generating research.

## Data Availability

The raw data supporting the conclusions of this article will be made available by the authors, without undue reservation.

## References

[1] Eyles DW Burne TH McGrath JJ . Vitamin D, effects on brain development, adult brain function and the links to neuropsychiatric disease. Front Neuroendocrinol. (2013) 34:47–64. 10.1016/j.yfrne.2012.07.001 22796576

[2] Groves NJ McGrath JJ Burne TH . Vitamin D as a neurosteroid affecting the developing and adult brain. Annu Rev Nutr. (2014) 34:117–41. 10.1146/annurev-nutr-071813-105557 25033060

[3] Whitehouse AJ Holt BJ Serralha M Holt PG Kusel MM Hart PH . Maternal serum vitamin D levels during pregnancy and offspring neurocognitive development. Pediatrics. (2012) 129:485–93. 10.1542/peds.2011-2644 22331333

[4] Daraki V Roumeliotaki T Koutra K Georgiou V Chalkiadaki G Karachaliou M . High maternal vitamin D levels during pregnancy may protect against behavioral difficulties in preschoolers. Nutrients. (2018) 10:1455. 30297599

[5] Voltas N Canals J Hernández-Martínez C . Maternal vitamin D status during pregnancy and child neurodevelopment: a systematic review. Nutr Rev. (2021) 79:658–71.

[6] Jiang X Stewart P Rodriguez A Brown AS Cheslack-Postava K Öhman H . Maternal vitamin D and offspring cognitive development: updated systematic review and meta-analysis. Nutrients. (2023) 15:212. 36615872

[7] Cannell JJ . Vitamin D and autism, what's new?. Rev Endocr Metab Disord. (2017) 18:183–93. 10.1007/s11154-017-9409-0 28217829

[8] Karras SN Shah I Petroczi A Goulis DG Bili H Papadopoulou F . An observational study reveals that neonatal vitamin D is primarily determined by maternal contributions: implications of a new assay on the roles of vitamin D forms. Nutrition Journal. (2013) 12:77. 23911222 10.1186/1475-2891-12-77 PMC3680300

[9] Karras SN Koufakis T Antonopoulou V Goulis DG Annweiler C Pilz S . Characterizing neonatal vitamin D deficiency in the modern era: a maternal–neonatal birth cohort from Southern Europe. J Steroid Biochem Mol Biol. (2020) 198:105555. 31783152 10.1016/j.jsbmb.2019.105555

[10] van Rooij D Mou Y White T Voortman T Jansen PW Buitelaar JK . Prenatal Vitamin D, Multivitamin, and Folic Acid Supplementation and Brain Structure in Children with ADHD and ASD Traits: The Generation R Study. Nutrients. (2025) 17:2979. 41010504 10.3390/nu17182979 PMC12472298

[11] Arrhenius B Upadhyaya S Hinkka-Yli-Salomäki S Brown AS Cheslack-Postava K Öhman H . Prenatal Vitamin D Levels in Maternal Sera and Offspring Specific Learning Disorders. Nutrients. (2021) 13:3321. 34684323 10.3390/nu13103321 PMC8539854

[12] Sass L Vinding RK Stokholm J Bjarnadóttir E Noergaard S Thorsen J . High-Dose Vitamin D Supplementation in Pregnancy and Neurodevelopment in Childhood: A Prespecified Secondary Analysis of a Randomized Clinical Trial. JAMA Netw Open. (2020) 3:e2026018. 33289844 10.1001/jamanetworkopen.2020.26018 PMC7724557

[13] Brouwer-Brolsma EM Vrijkotte TGM Feskens EJM . Maternal vitamin D concentrations are associated with faster childhood reaction time and response speed, but not with motor fluency and flexibility, at the age of 5-6 years: the Amsterdam Born Children and their Development (ABCD) Study. Br J Nutr. (2018) 120:345–52. 10.1017/S0007114518001319 29843832

[14] Vinkhuyzen AAE Eyles DW Burne TH Blanken LME Kruithof CJ Verhulst F . Prevalence and predictors of vitamin D deficiency based on maternal mid-gestation and neonatal cord bloods: The Generation R Study. J Steroid Biochem Mol Biol. (2016) 164:161–67. 10.1016/j.jsbmb.2015.09.018 26385604

[15] Strøm M Halldorsson TI Hansen S Granström C Maslova E Petersen SB . Vitamin D measured in maternal serum and offspring neurodevelopment: a prospective study with long follow-up. Eur J Clin Nutr. (2014) 68:1022–8. 10.1159/000365030 25300268

[16] Voltas N Cendra-Duarte E Canals J Arija V . Vitamin D status during pregnancy and child neurocognitive functioning at 4 Years. Pediatr Res. (2026) 99:534–43. doi: 10.1038/s41390-025-04258-9, PMID: 40731094

[17] Cantio E Bilenberg N Nørgaard SM Beck IH Möller S Cantio C . Vitamin D status in pregnancy and childhood associates with intelligence quotient at age 7 years: An Odense child cohort study. Aust N Z J Psychiatry. (2023) 57:1062–72. 10.1177/00048674221116027 35971641

[18] Thinggaard CM Dalgård C Möller S Christesen HBT Bilenberg N . Vitamin D status in pregnancy and cord blood is associated with symptoms of attention-deficit hyperactivity disorder at age 5 years: Results from Odense Child Cohort. Aust N Z J Psychiatry. (2024) 58:1090–102. 10.1177/00048674241272018 39152569

[19] Autier P Boniol M Pizot C Mullie P . Vitamin D status and ill health: A systematic review. Lancet Diabetes Endocrinol. (2014) 2:76–89. 10.1016/S2213-8587(13)70165-7 24622671

[20] Aranow C . Vitamin D and the immune system. J Investig Med. (2011) 59:881–6. 10.231/JIM.0b013e31821b8755 PMC3166406 21527855

[21] Gupta A Bansal R Gupta V Goyal S . Vitamin D deficiency and disease correlation. Int J Med Sci Public Health. (2014) 3:1056–9.

[22] Holick MF Mazzei L García Menéndez S Martín Giménez VM Al Anouti F Manucha W . Genomic or non-genomic? A question about the pleiotropic roles of vitamin D in inflammatory-based diseases. Nutrients. (2023) 15:767. 36771473 10.3390/nu15030767 PMC9920355

[23] Karras SN Kotsa K Angeloudi E Zebekakis P Naughton DP . The Road Not So Travelled: Should Measurement of Vitamin D Epimers during Pregnancy Affect Our Clinical Decisions?. Nutrients. (2017) 9:90. 28134839 10.3390/nu9020090 PMC5331521

[24] Vierucci F Fusani L Saba A Minucciani T Belluomini MP Domenici R . Gestational vitamin D3 supplementation and sun exposure significantly influence cord blood vitamin D status and 3-epi-25-hydroxyvitamin D3 levels in term newborns. Clin Chim Acta. (2022) 524:59–68. 10.1016/j.cca.2021.11.022 34838794

[25] Mydtskov ND Lykkedegn S Fruekilde PBN Nielsen J Barington T Christesen HT . Clin Biochem. Clin Biochem. (2017) 50:988–96. 10.1016/j.clinbiochem.2017.07.001 28697996

[26] Park H Wood MR Malysheva OV Jones S Mehta S Brannon PM . Placental vitamin D metabolism and its associations with circulating vitamin D metabolites in pregnant women. Am J Clin Nutr. (2017) 106:1439–48. 10.3945/ajcn.117.153429 PMC5698837 29021285

[27] Mendoza LC Harreiter J Desoye G Simmons D Adelantado JM Kautzky-Willer A . The Weak Relationship between Vitamin D Compounds and Glucose Homeostasis Measures in Pregnant Women with Obesity: An Exploratory Sub-Analysis of the DALI Study. Nutrients. (2022) 14:3256. 36014761 10.3390/nu14163256 PMC9415540

